# Evaluation of [^68^Ga]DO3A-VS-Cys^40^-Exendin-4 as a PET Probe for Imaging Human Transplanted Islets in the Liver

**DOI:** 10.1038/s41598-019-42172-3

**Published:** 2019-04-05

**Authors:** Junfeng Li, Jeffrey Rawson, Junie Chea, Wei Tang, Lynn Miao, Feng Sui, Lin Li, Erasmus Poku, John E. Shively, Fouad Kandeel

**Affiliations:** 1 Department of Translational Research & Cellular Therapeutics, Beckman Research Institute of the City of Hope, Duarte, USA; 2 Department of Molecular Imaging and Therapy, Beckman Research Institute of the City of Hope, Duarte, USA

## Abstract

[^68^Ga]DO3A-VS-Cys^40^-Exendin-4, a glucagon-like peptide 1 receptor agonist, was evaluated as a potential PET tracer for the quantitation of human islets transplanted to the liver. The short-lived PET radionuclide ^68^Ga, available on a regular basis from a ^68^Ge/^68^Ga generator, is an attractive choice. Human C-peptide was measured to evaluate human islet function post-transplantation and prior to microPET imaging. [^68^Ga]DO3A-VS-Cys^40^-Exendin-4 was radiosynthesized and evaluated for PET imaging of transplanted human islets in the liver of healthy NOD/SCID mice. The biodistribution of the tracer was evaluated to determine the uptake into various organs, and qPCR of liver samples was conducted to confirm engrafted islet numbers after PET imaging. Measurement of human C-peptide indicated that higher engrafted islet mass resulted in higher human C-peptide levels in post-transplantation. The microPET imaging yielded high resolution images of liver-engrafted islets and also showed significant retention in mouse livers at 8 weeks post-transplantation. Biodistribution studies in mice revealed that liver uptake of [^68^Ga]DO3A-VS-Cys^40^-Exendin-4 was approximately 6-fold higher in mice that received 1000 islet equivalent (IEQ) than in non-transplanted mice. qPCR analysis of insulin expression suggested that islet engraftment numbers were close to 1000 IEQ transplanted. In conclusion, human islets transplanted into the livers of mice exhibited significant uptake of [^68^Ga]DO3A-VS-Cys^40^-Exendin-4 compared to the livers of untreated mice; and imaging of the mice using PET showed the human islets clearly with high contrast against liver tissue, enabling accurate quantitation of islet mass. Further validation of [^68^Ga]DO3A-VS-Cys^40^-Exendin-4 as an islet imaging probe for future clinical application is ongoing.

## Introduction

Pancreatic islet transplantation is a minimally invasive procedure that can restore normal blood sugar levels and insulin independence in type 1 diabetes (T1D) patients^1^. Islet transplantation may prevent or even reverse T1D complications, as well as improve quality of life through the reduction or elimination of hypoglycemia and/or hypoglycemia unawareness. Despite its great therapeutic potential, however, to date, ~50% of patients have experienced loss of transplanted islets within 5 years of transplantation^2–5^.

Due to a lack of suitable tools for the *in vivo* monitoring of islets post-transplantation, evaluation of their survival and function is limited to indirect metabolic assessments, such as the glucose tolerance test (GTT), mixed meal tolerance test (MMT), and glucose-potentiated arginine test. These tests are inadequate and can impede the assessment of islet cells in the early stages post-transplantation for several reasons including: (1) that auto-islets have four- to five-fold greater functional capacity post-transplantation than the same number of allogeneic islets^6,7^; and (2) in the case of hyperglycemia, a small number of islets may produce excess glucose and thus would not accurately reflect islet mass. Therefore, it is necessary to develop an *in situ* technique to directly monitor islet mass and function.

Positron emission tomography (PET) is a molecular imaging technique that permits noninvasive measurement of tracer disposition and localization in organs and tissues *in vivo*^8–11^. It is an important tool for preclinical and clinical studies in oncology, neuroimaging and diabetes^12–16^ and has the potential to provide quantitative analysis of transplanted islets in the liver, as well as an estimate of how many islets remain alive and functional over time. This information would likely lead to a better understanding of why some islet transplants are successful while others are not. Furthermore, this strategy would facilitate the development of therapies to prolong islet transplant survival in T1D patients on a widespread and long-term basis.

However, challenges remain in developing PET tracers to image transplanted human islets in the liver. Proteins such as VMAT2^17,18^, SUR1^19^, and the D2 receptor^20^, have been identified as biomarkers for native islets and corresponding PET tracers have been developed for imaging islets in the pancreas. Unfortunately, most tracers developed for the pancreas are not suitable for monitoring viable islet mass after clinical intraportal liver islet transplantation, as some tracers are metabolized in the liver resulting in significant background signal in hepatic grafts^21^; and other tracers exhibit high levels of non-specific binding in the liver.

In our previous studies, we identified glucagon-like peptide 1 receptor (GLP-1R) as an ideal biomarker for imaging islets and insulinomas^22–24^. Activated GLP-1R stimulates the adenylyl cyclase pathway, resulting in increased insulin synthesis and release. Because GLP-1R is known to be expressed in pancreatic beta cells with low expression in the liver^25,26^, we reasoned that the target-to-background contrast would be suitable for imaging transplanted islets in the liver. Exendin-4 as a GLP-1R agonist, was first isolated from the saliva of the Gila monster in 1992^27^. It displays biological properties similar to human GLP-1, with which it shares 53% sequence identity^28^; however, it is metabolically more stable than GLP-1^29,30^. To date, only a few PET tracers based on an exendin-4 fragment have been developed and evaluated in insulinoma models^31–33^. However, they have not been evaluated for imaging transplanted human islets in the liver for several reasons. First, transplanted pancreatic islets from human donors pancreastas can be maintained for less than one week without loss of islet mass and function. Second, intraportal transplantation of human islets into the livers of small animals is technically challenging. Therefore, to our knowledge, only the exendin-4 PET tracers developed by our team have been evaluated for imaging pancreatic human islets in mouse livers^22,23^. Although the tracers ^18^F-TTCO-Cys^40^-Exendin-4 and ^64^Cu-DO3A-VS-Cys^40^-Exendin-4 displayed highly specific binding to GLP-1R in the mouse insulinoma model, they did not provide sufficient contrast for PET islet imaging in a mouse model of human islet transplantation. Hepatic uptake of both tracers in mice transplanted with 1000 IEQ was only about two-fold higher than that in mice that received a mock transplant^22,23^. We attribute this discrepancy to the fact that GLP-1R is overexpressed nearly five-fold in insulinoma compared to normal human β-cells^34^ and that intraportally transplanted human islets do not distribute homogeneously throughout the liver. Therefore, it is necessary to develop a more suitable PET tracer to improve islet imaging in the liver.

Previously, we and our collaborators reported that [^68^Ga]DO3A-VS-Cys^40^-Exendin-4 as a PET tracer was able to detect GLP-1R in insulinomas^35–37^. Further, we also found that [^68^Ga]DO3A-VS-Cys^40^-Exendin-4 had low uptake in sham operated liver, supporting our hypothesis that the low background signal in the liver would not interfere with detection of a transplanted islet mass. Therefore, the present study further evaluated [^68^Ga]DO3A-VS-Cys^40^-Exendin-4 as a PET tracer for imaging human islets in livers of NOD/SCID mice.

## Experiment

### Radiochemistry

^68^Ga was obtained from a bench top ^68^Ge/^68^Ga generator system (1850 MBq, Eckert & Ziegler, IGG 100). Elution was performed with 0.1 M HCl. The first 1.5 mL fraction was discarded and the next 3.0 mL containing over 90% of the total radioactivity, was collected in a glass vial containing 10.5 nmol DO3A-VS-Cys^40^-Exendin-4 buffered with 0.6 mL of 1 M ammonium acetate buffer, 0.05 mL plasma grade water and 0.2 mL ethanol at a final pH of 4.6 ± 0.1. The mixture was incubated at 75 °C for 15 min. The final product, [^68^Ga]DO3A-VS-Cys^40^-Exendin-4, was formulated in 1% human serum albumin (HSA) in phosphate buffered saline (PBS) to yield a pH of 7.4.

### Animal model

Male NOD/SCID mice (n = 7 per group, 8–10 weeks old, City of Hope Animal Resource Center) served as recipients of human islets. The mice were maintained and used according to the institutional guidelines of the City of Hope/Beckman Research Institute’s Institutional Animal Care and Use Committee (IACUC). All animal experiments were conducted in compliance with the protocols approved by the Care and Use of Research Animals established by the City of Hope/Beckman Research Institute’s Institutional Animal Care and Use Committee (IACUC#15035, Approved 7/27/17).

Human pancreatic islets with a diameter of less than 250 µm were transplanted into the livers of NOD/SCID mice via the portal vein. Immediately before intraportal infusion, 500 or 1000 IEQ islets were suspended in 0.1 mL of 1x transfer medium and loaded in a 1 mL insulin syringe. Under general anesthesia, the portal vein was exposed by extra-abdominal repositioning of the bowel. Islets were then infused into the portal vein via a 27.5 gauge needle.

### Human C-peptide measure

Overnight fasted NOD/SCID mice underwent blood collection from the retro-orbital plexus at 1, 3, 5, and 8 weeks post-transplantation. After centrifugation of the blood samples at 6,500 rpm for 10 min, serum in the supernatant was collected for human C-peptide quantification using a Mercodia C-peptide ELISA kit (Mercodia, 10-1136-01).

### MicroPET imaging

MicroPET imaging of [^68^Ga]DO3A-VS-Cys^40^-Exendin-4 was performed to measure islets in the livers of the mice that also underwent human C-peptide quantification at 8 weeks post-transplantation. Mice were anesthetized with 2–4% isoflurane in oxygen before intravenous injections via the tail of ~4 MBq [^68^Ga]DO3A-VS-Cys^40^-Exendin-4 in 1% HSA with PBS. MicroPET imaging was conducted at 90 min time points using a Siemens Inveon microPET system to acquire whole body PET imaging. Kidneys were removed prior to microPET imaging. The livers and pancreata were collected to scan in microPET scanner.

### Biodistribution of [^68^Ga]DO3A-VS-Cys^40^-Exendin-4 in mice

The biodistribution of [^68^Ga]DO3A-VS-Cys^40^-Exendin-4 was investigated in NOD/SCID healthy mice with and without human islets in the liver. NOD/SCID mice were administered doses of tracer intravenously through the tail vein under general anesthesia, as described above. At 90 min post-injection, mice were anesthetized and euthanized. After mciroPET imaging, blood, lung, heart, liver, spleen, stomach, small intestine, large intestine, muscle, kidney, pancreas and bone were collected and weighed, and radioactivity was counted. Results were reported as percentage injected dose per gram (%ID/g).

### TaqMan real-time quantitative PCR (qPCR)

Total RNA was extracted from serially diluted, isolated human islets mixed with whole mouse livers or whole mouse livers transplanted with human islets using a Direct-zol RNA MicroPrep Kit from ZYMO Research (Cat# R2060), per the manufacturer’s instructions. 1 µg of RNA from each sample was used to synthesize cDNA with a Superscript II First-Strand cDNA Synthesis kit (Invitrogen Inc.) with oligo (dT) primers. The cDNA was analyzed with TaqMan real-time qPCR using TaqMan Universal qPCR Master Mix in an AB 7500 fast real-time system (Applied Biosystems). Briefly, a 25 μL reaction mixture consisted of 12.5 μL TaqMan Universal qPCR Master mix, 400 nM primers (Applied Biosystems), and 300 nM TaqMan probe (Applied Biosystems) for each of the target genes. Thermal cycle conditions were 95 °C for 2 min (*Taq activation*), 95 °C for 15 seconds for denaturation, and 60 °C for 1 min for annealing and extension.

### Immunohistochemistry

Mouse livers used for PET and biodistribution studies (after mock transplantation or transplantation with 1000 IEQ islets) were fixed with 10% formalin for 24 hours. The sections (50 µM) were stained by immunochemistry using primary antibodies (Guinea pig anti-human insulin IgG antibody; Mouse anti-glucagon IgG antibody) and secondary antibodies (Cy3 donkey anti-Guinea pig IgG; Alexa Fluor 488 Donkey anti-mouse IgG). The stained sections were mounted on slides and visualized with an LSM 700 confocal laser scanning microscope.

## Results

### Radiochemistry

**Figure 1:**
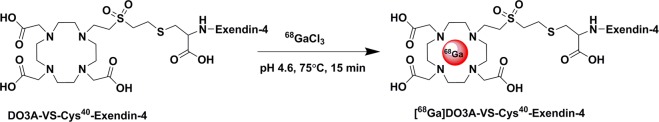
Schematic representation of [^68^Ga]DO3A-VS-Cys^40^-Exendin-4 radiosynthesis.

^68^GaCl_3_ (925-1110 MBq) was obtained from a ^68^Ge/^68^Ga generator system. Eluted ^68^GaCl_3_ was reacted with DO3A-VS-Cys^40^-Exendin-4 to produce [^68^Ga] DO3A-VS-Cys^40^-Exendin-4. Medium radiochemical yield (about ~70%, decay corrected), high radiochemical purity (>99%), and high specific activity (>51 GBq/µmol) were achieved with a total synthesis time of 30 min (Fig. 1).

### Human C-peptide levels in NOD/SCID mice transplanted with human islets

**Figure 2:**
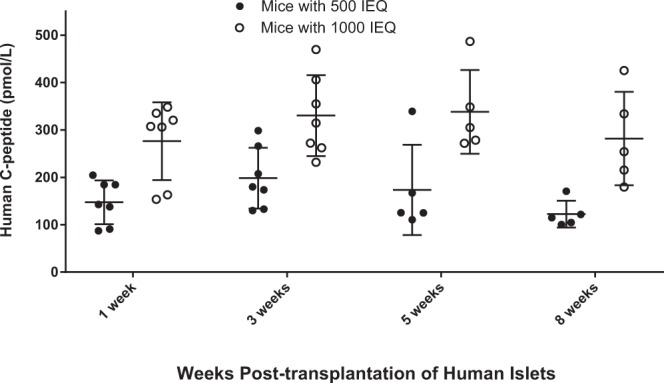
Human C-peptide levels in NOD/SCID healthy mice measured at 1, 3, 5, and 8 weeks post-transplantation of islets to the liver. The levels of human C-peptide in mice with 1000 IEQ is approximately two-fold that of mice with 500 IEQ across all time points.

The levels of human C-peptide were measured in the healthy NOD/SCID mice transplanted with 0, 500 and 1000 IEQ before PET studies (Fig. 2). Human C-peptide levels were not detected in mock transplanted mice (data not shown). Human C-peptide levels in mice that received 500 IEQ were 147.4 ± 46.3, 198.3 ± 64.0, 173.5 ± 95.0, and 122.4 ± 28.3 pmol/L at 1, 3, 5, and 8 weeks post-transplantation, respectively. For mice that received 1000 IEQ, values were 276.3 ± 81.9, 330 ± 85.3, 338.0 ± 85.3, and 281.7 ± 98.6 pmol/L, respectively. Human C-peptide levels in mice with 1000 IEQ were about two-fold higher than in mice with 500 IEQ. That is, higher islet mass resulted in higher human C-peptide levels.

### Small animal PET imaging studies of NOD/SCID mice transplanted with human islets

**Figure 3:**
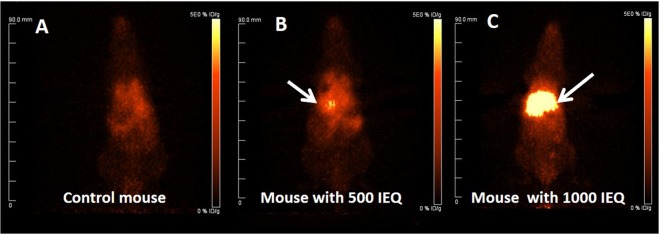
Representative coronal maximum intensity projection small animal PET images at 90 min p.i. (**A**) Control NOD/SCID mouse. (**B** and **C**) NOD/SCID mice with human islets (500 and 1000 IEQ, respectively). Kidneys were removed before microPET imaging. The liver with transplanted islets demonstrate prominent tracer uptake (arrow).

Whole-body PET images were obtained for [^68^Ga]DO3A-VS-Cys^40^-Exendin-4 in NOD/SCID mice (control, 500 and 1000 IEQ at 8 weeks post-transplantation, respectively). Representative whole-body PET imaging for NOD/SCID mice without islets, and with 500 and 1000 IEQ islets are shown in Fig. 3. Notably, mice with 1000 IEQ had significant uptake in the liver, native pancreas and lung, demonstrating clear visualization of the GLP-1R-enriched islet mass in the liver and in two normal tissues expressing GLP-1R. As expected, we were able to detect the tracer in the native pancreas with no difference in uptake between transplanted groups.

### Biodistribution in NOD/SCID mice with or without human islets

**Figure 4:**
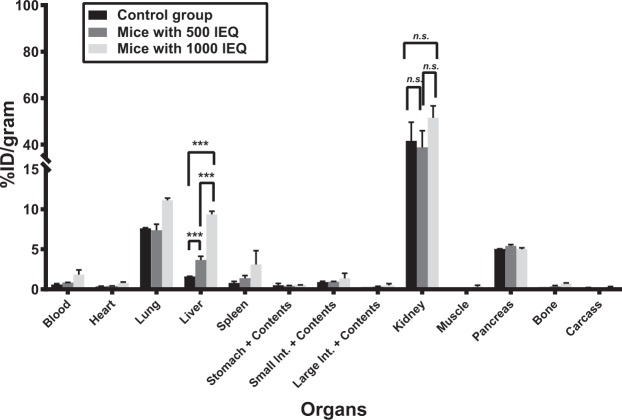
The biodistribution of [^68^Ga]DO3A-VS-Cys40-Exendin-4 was evaluated in mice across various tissues. The percentage injected dose per gram tissue (%ID/g) is shown for the control group and for mice that received 500 or 1000 IEQ islets (Student *t* test, ***p < 0.001, *n.s*: not significant).

The biodistribution of [^68^Ga]DO3A-VS-Cys^40^-Exendin-4 was evaluated in the livers of NOD/SCID male mice with or without human islets at 90 min post-injection (Fig. 4**)**. The uptake values for the liver were 1.60 ± 0.02%ID/g for the control group, and 3.67 ± 0.46 and 9.36 ± 0.39%ID/g for mice with 500 IEQ and 1000 IEQ, respectively. The uptake values of mice with 500 IEQ and 1000 IEQ were significantly higher than the uptake value of control group mice (p < 0.05 for both comparisons). Notably, the hepatic uptake of tracer in mice that received 1000 IEQ *vs*. in control livers was approximately 6-fold higher. Analysis of the pancreas as a control GLP-1R-positive organ revealed uptake values of 5.05 ± 0.01, 5.44 ± 0.14, and 5.00 ± 0.19%ID/g for the control, 500 IEQ, and 1000 IEQ groups, respectively, with no significant differences between the control and treatment groups (p > 0.05). Progressive accumulation of radioactivity in kidneys was observed as expected for small peptide excretion. The uptake values for the kidney were 41.6 ± 8.01, 38.85 ± 7.12, and 51.71 ± 5.10%ID/gram for the control, 500 IEQ, and 1000 IEQ groups, respectively, with no significant differences between the control and treatment groups (p > 0.05). These result confirm that the tracer is cleared via kidney uptake and excretion.

Taken together, the biodistribution data agreed with the microPET imaging results.

### qPCR analysis

**Figure 5:**
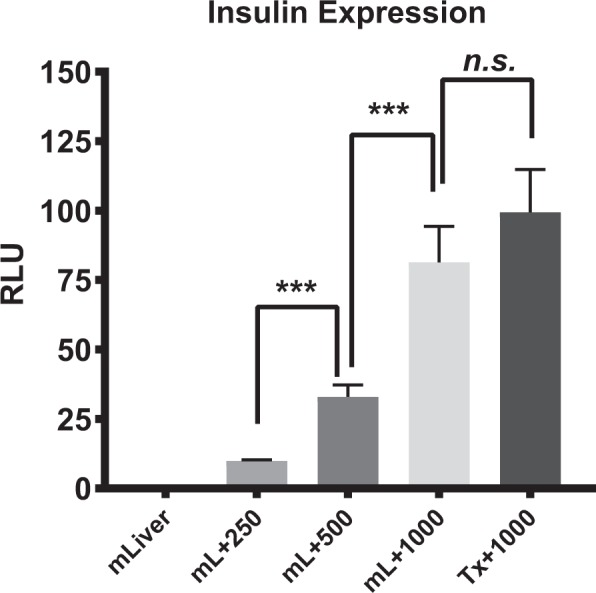
Insulin expression in mixtures of mouse liver with various amounts of human islets (0, 250, 500, 1000 IEQ) and whole liver after human islet transplantation (1000 IEQ). Insulin was not detected in control livers (mLiver). For the livers mixed with islets (mL + 250, mL + 500, and mL + 1000), greater islet numbers resulted in higher insulin expression. Insulin expression in the livers with engrafted islets was close to that of the mL + 1000 group. Expression levels were compared between groups using unpaired Student’s tests. n.s: not significant (*p* = 0.19); ***p < 0.001. mLiver = whole liver without islets, mL + 250 = whole liver + 250 IEQ; mL + 500 = whole liver + 500 IEQ; mL + 1000 = whole liver + 1000 IEQ; Tx + 1000 = whole liver after transplantation of 1000 IEQ.

In order to estimate the number of transplanted islets in the liver by qPCR, we first established the relationship between insulin expression levels and the number of isolated human islets (0, 250, 500, and 1000 IEQ) mixed with mouse liver (Fig. 5). In the control group (0 IEQ), insulin expression could not be detected. For liver tissues mixed with 250, 500, and 1000 IEQ, insulin express levels were 9.83 ± 0.46, 32.98 ± 4.20, and 81.35 ± 12.90, respectively. These results demonstrate that a higher IEQ corresponds with higher insulin expression as measured by qPCR. Next, we collected livers transplanted with 1000 IEQ and measured insulin expression. There were no significant differences between insulin expression in whole livers mixed with 1000 IEQ and those engrafted with 1000 IEQ.

### Immunohistology

**Figure 6:**
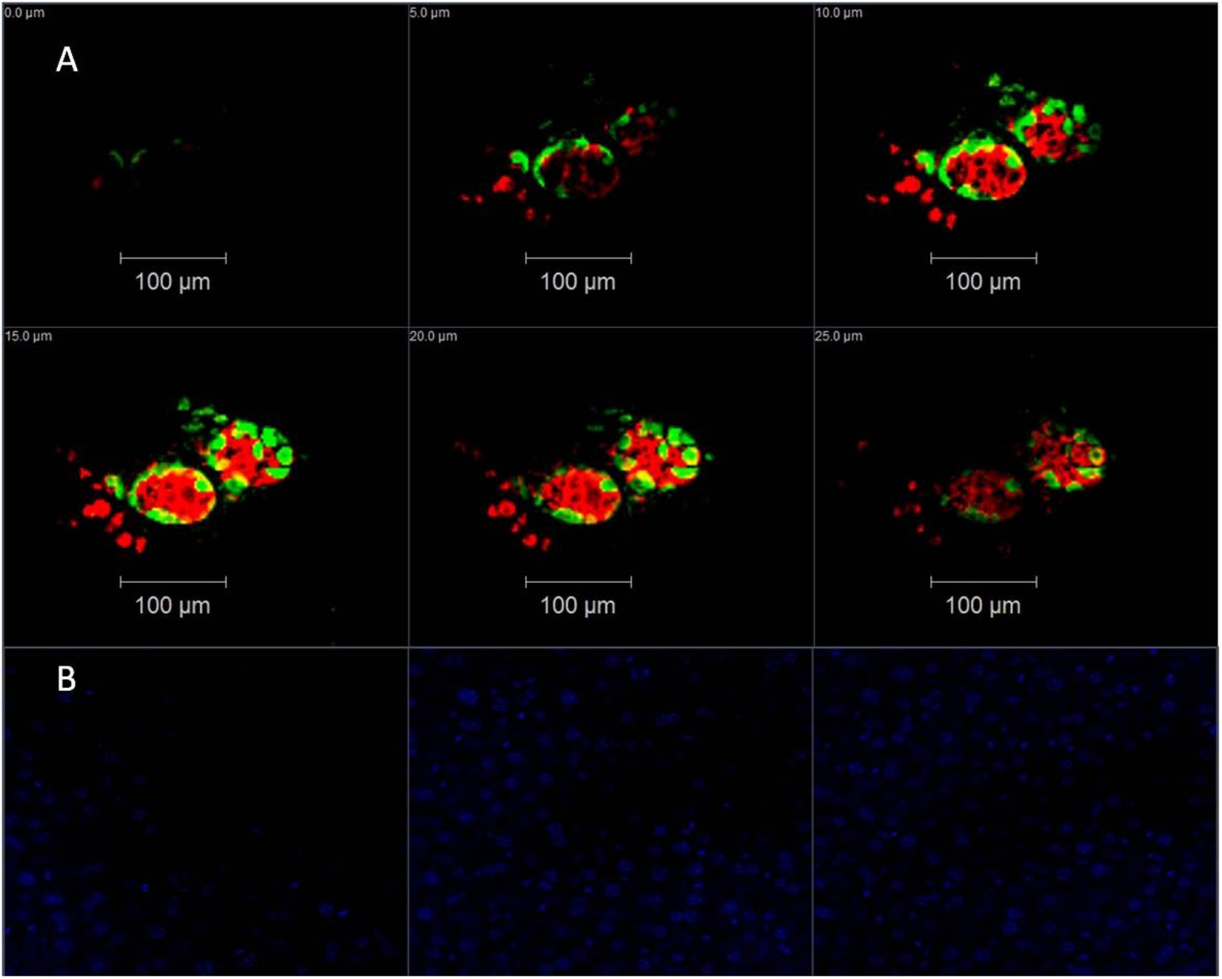
Representative fluorescent confocal microscopy images of NOD/SCID mouse liver with/without engrafted human islets. Tissues were stained with anti-insulin antibodies (red), anti-glucagon antibodies (green), and DAPI (blue). A series of optical sections was acquired at 5-µm intervals through the liver slides in the axial (z) dimension. (**A**) Mouse liver engrafted with human islets. (**B**) Control liver without islets. Only DAPI is displayed.

Following microPET and biodistribution studies, murine livers were collected for immunohistochemistry staining. We compared insulin (beta cell) and glucagon (alpha cell) expression levels in mock transplanted livers and in the livers of mice transplanted with 1000 IEQ. Our data demonstrated that human islets were clearly present in the livers of mice 8 weeks after transplantation but not in mock transplanted livers (Fig. 6). These data are consistent with the microPET and biodistribution results.

## Discussion

In this study, we assessed the use of [^68^Ga]DO3A-VS-Cys^40^-Exendin-4 to monitor islets post-transplantation into mouse livers. Overall, the tracer was highly sensitive, with minimal background expression in control livers. Image resolution was also quite high, enabling clear localization of the islet mass.

There are several rodent models available for assessment of PET islet imaging tracers: (1) syngeneic rodent islets transplanted to the liver; (2) syngeneic rodent islets transplanted to an intramuscular site; and (3) native islets in the rodent pancreas. However, there are several drawbacks to each of these models. First, the cytoarchitecture of human compared to rodent pancreatic islets differs^38–40^. In particular, whereas rodent islets are characterized by a predominant proportion of insulin-producing beta cells in the core of the cluster with scarce alpha, delta and pancreatic polypeptide cells in the periphery, human islets display closely associated alpha and beta cells distributed throughout the islet cluster. Second, pancreatic islets transplanted to the liver are relatively small in size and few in number compared to the whole liver. Furthermore, they do not distribute homogeneously throughout the liver. By contrast, islets in the pancreas or transplanted into muscle tend to be more evenly distributed at higher concentrations. Therefore, although tracers may be effective for imaging islets in the pancreas and muscle, these tracers may not be appropriate for imaging islets transplanted to the liver. Models 2 and 3 are sufficient ways to evaluate these tracers in the pancreas and muscle but not in the liver. Third, the cellular environment in the liver differs from that in muscle and pancreas. For example, the tracers that have been successful for imaging islets in muscle and pancreas are metabolized and/or have greater non-specific binding in the liver, resulting in high background signals. Therefore, in order to develop a model more relevant to the clinical setting, we used an intraportal human islet transplant model in mouse livers, along with clinical-grade human islets to evaluate these PET tracers.

In rats, a very low number (about 550–600) of syngeneic intra-portal islets is sufficient to reverse diabetes^41^. Since transplantation to the liver requires the smallest number of islets for reversal of hyperglycemia, it has been presumed to be the most hospitable and has become the standard for comparison with other potential implantation locations. In our current study, 500 and 1000 IEQ from human donors were transplanted into the livers of healthy NOD/SCID mice. We confirmed the efficacy of the transplantations by measuring human C-peptide levels in the sera of mice. As expected, human C-peptide levels were greatest for mice that received 1000 IEQ.

We previously reported that ^18^F-TTCO-Cys^40^-Exendin-4^22^ and ^64^Cu-DO3A-VS-Cys^40^-Exendin-4^23^ can detect human islets in the livers of rodents. However, these tracers exhibited limited potency and sensitivity. The uptake of ^18^F-TTCO-Cys^40^-Exendin-4 in the liver of transplanted mice (1000 IEQ) was higher than uptake in the livers of control mice, but the difference was only about 1.88–2.80 fold. Uptake of ^64^Cu-DO3A-VS-Cys^40^-Exendin-4, in the liver was 5.3 ± 0.7%ID/g at 5 h post injection for mice that received islet transplantation, which was only slightly (2.2-fold) greater than uptake in mice that received a mock transplant (2.2 fold, 1000 IEQ). Obtaining a high target-to-liver ratio is critical for noninvasive *in vivo* PET imaging of islets transplanted in the liver because of the small size and number of pancreatic islets engrafted. In our current study using [^68^Ga]DO3A-VS-Cys^40^-Exendin-4, uptake in the livers of mice with 1000 IEQ was about 6-fold greater than that found in livers in the mock transplantation group, resulting in a high target-to-liver contrast in PET imaging. Immunohistological and qPCR analyses were also performed, and the insulin levels and islet mass numbers estimated were consistent with the results of our microPET and biodistribution studies.

The use of [^68^Ga]DO3A-VS-Cys^40^-Exendin-4 as a PET tracer for imaging transplanted islets is not only more effective but also more convenient than previously developed tracers. The PET radionuclide ^68^Ga can be produced by a ^68^Ge/^68^Ga generator, which serves as an instant source of the short-lived radionuclide. Whereas the production of other tracers might require an onsite cyclotron, medical grade ^68^Ge/^68^Ga generators are widely available, making it possible to produce [^68^Ga]DO3A-VS-Cys^40^-Exendin-4 in any clinic with access to a Nuclear Medicine department.

A possible limitation of using [^68^Ga]DO3A-VS-Cys^40^-Exendin-4 as a tracer is that retention is high in the kidney cortex, which is similar to retention of ^64^Cu^42^, ^99m^TC^43^ and ^111^In^44^ in other studies. This high uptake may ultimately limit its clinical application due to radiation exposure to this radiosensitive organ. However, some encouraging results were reported^37^ in the dosimetry of [^68^Ga]DO3A-VS-Cys^40^-Exendin-4 based on biodistribution data from rats, pigs, and non-human primates. The absorbed dose to the kidneys was the dose limiting factor, and based on data from these species, ~0.28–0.65 mGy/MBq, which corresponds to maximum yearly administered amounts of ~231–536 MBq, can be administered before reaching the yearly kidney limiting dose of 150 mGy. Therefore, it was estimated that >200 MBq [^68^Ga]DO3A-VS-Cys^40^-Exendin-4 can be administered yearly, allowing for repeated scanning (2–4 times per year) in humans. This could enable longitudinal clinical PET imaging studies of the GLP-1R in transplanted islets.

## Conclusion

We transplanted human islets into the livers of mice and imaged them at 8 weeks post-transplantation. MicroPET imaging of [^68^Ga]DO3A-VS-Cys^40^-Exendin-4 produced high contrast images of the islets, and our biodistribution study revealed that tracer uptake in the livers of mice transplanted with 1000 IEQ was about 6-fold that in the livers of mock transplanted mice. To our knowledge, this is the highest ratio reported for tracers used to image beta cells in the liver. Human C-peptide measurements, qPCR, and histological analyses further confirmed the numbers and function of transplanted islets. Taken together, these data demonstrate that [^68^Ga]DO3A-VS-Cys^40^-Exendin-4 is highly sensitive and can produce significant contrast for imaging human islets in the liver. Further validation of [^68^Ga]DO3A-VS-Cys^40^-Exendin-4 as a suitable PET tracer to quantify human islet mass and function in the liver for clinical applications is ongoing.

## Data Availability

The datasets generated and/or analysed during the current study are available from the corresponding author on reasonable request.
